# First record of the symbiotic palaemonid shrimp *Pontonidessibogae* Bruce, 2005 (Crustacea, Decapoda, Palaemonidae) from Korea

**DOI:** 10.3897/BDJ.10.e85913

**Published:** 2022-06-16

**Authors:** Jin-Ho Park, Sammy De Grave, Taeseo Park

**Affiliations:** 1 Computational Biology Institute, The George Washington University, Washington, DC, United States of America Computational Biology Institute, The George Washington University Washington, DC United States of America; 2 Oxford University Museum of Natural History, Oxford, United Kingdom Oxford University Museum of Natural History Oxford United Kingdom; 3 National Institute of Biological Resources, Incheon, Republic of Korea National Institute of Biological Resources Incheon Republic of Korea

**Keywords:** Caridea, Palaemonidae, *Pontonidessibogae*, dendrophylliid coral symbiont, new record, Korea

## Abstract

**Background:**

A single specimen of *Pontonidessibogae* was collected from a dendrophylliid coral by trimix SCUBA diving at a depth of 75 m during fieldwork around Jejudo Island, Korea in 2020. The morphology of the specimen corresponds closely to the main diagnostic characters of the holotype, especially in the presence of a distinct tubercle on the eyestalk, as well as the second pereiopod with fusiform setae along the dorsal border of the ischium.

**New information:**

The genus *Pontonides* had previously not been reported from Korean waters. Herein, colour photographs are provided, as well as an illustrated description including previously unreported characteristics.

## Introduction

The genus Pontonides Borradaile, 1917 currently comprises seven hexacoral-associated species from tropical to temperate waters on the Indo-West Pacific: *Pontonidesmaldivensis* (Borradaile, 1915) (type species); *P.unciger* Calman, 1939; *P.asperulatus* Bruce, 2005; *P.loloata* Bruce, 2005; *P.sibogae* Bruce, 2005; *P.ankeri* Marin, 2007; and *P.tatianae* Marin, 2007 (Borradaile 1915, Borradaile 1917, Calman 1939, Bruce 2005, Marin 2007, De Grave and Fransen 2011). The genus is well known for its cryptic colour pattern, mimicking the background colour pattern of their host species (Marin 2007).

Holthuis (1952) reported upon a single specimen of *Pontonidesunciger* which was collected by a dredge in a depth of 70 m from the Sape Strait in Indonesia during the Siboga Expedition in 1899. He provided a detailed description with illustrations of the lateral habitus, mouthparts, the first three pereiopods as well as the fusiform setae on the ischium of the second pereiopod. Fujino and Miyake (1969) reported upon ten specimens of *P.unciger*, which were collected from the branches of the scleractinian coral *Dendrophylliaijimai* Eguchi, 1965 collected by fishing gill nets from the Amakusa Islands, Japan, between 55 and 75 m. They described and illustrated sexual dimorphism and variation in the second pereiopods, as well as the presence of fusiform setae on the ischium of the second pereiopods. Heard (1986), when establishing the new genus *Pseudopontonides* Heard, 1986, suggested the existence of a *P.unciger* species complex, based on host specificity. Bruce (2005), when discussing previous records of *P.unciger*, established a new species (*P.sibogae*), based on the specimen assigned by Holthuis (1952) to *P.unciger*, using the presence of the tubercle on the eyestalk as a diagnostic character. The material described by Fujino and Miyake (1969) under the same name was also considered to belong to the new species. Marin (2007) provided several underwater photographs of Pontonidescf.sibogae on their host coral, *Dendrophyllia* sp., from an unspecified location in Japan. No further records of the species are known to date.

During fieldwork in the waters surrounding Jejudo Island, Korea in January 2020, a single specimen of *P.sibogae* was collected on a scleractinian dendrophylliid coral by trimix SCUBA diving at a depth from 75 m. In the present study, we recorded this species and indeed, the genus as a whole, as a new addition to the Korean fauna, including colour photographs, illustrations and a detailed description.

## Materials and methods

Faunal surveys around Jejudo Island, Korea were organised in 2020 jointly by Seoul National University (SNU), the Marine Biodiversity Institute of Korea (MABIK) and the National Institute of Biological Resources (NIBR). A single specimen of *Pontonidessibogae* was collected from the scleractinian dendrophylliid coral *Dendrophyllia* sp. with the aid of trimix diving at a depth of 75 m in Munseom Islet. Habitats were recorded using a digital camcorder (GoPro5; GoPro, US) in an underwater housing (DC-163Pro; Deepcube, Korea). Photographs of the specimen were taken using a digital camera (D850; Nikon, Japan) with a high-definition lens (Nikon AF-S VR Micro-Nikkor 105 mm f.28.G IF-ED; Nikon, Japan). Field collection number (fcn) was recorded and the sample preserved in 80% ethanol for morphological and molecular examinations. Morphological characteristics were observed using a stereomicroscope (M205C; Leica, Germany). Images for digital drawings were taken and postorbital carapace length (pocl, in mm) was measured from the postorbital margin to the posterior dorsal margin of the carapace using a microscope-mounted digital camera (MC170; Leica, Germany) and Leica software (Leica Acquire 3.45; Leica, Germany). Digital line drawings were made using Helicon focus software (Helicon focus 7.5.6, Ukraine) and a drawing tablet (Wacom Intuos Pro PTH-660, China) with Adobe Illustrator software (Adobe Systems, USA) following the method described by Coleman (2006). Material is deposited into NIBR, Incheon, Korea.

## Taxon treatments

### Pontonides sibogae

Bruce, 2005

B2660DC5-4BFD-58A7-87AA-1BADFC75BC9A

https://www.marinespecies.org/aphia.php?p=taxdetails&id=389607

**Figure 1.**
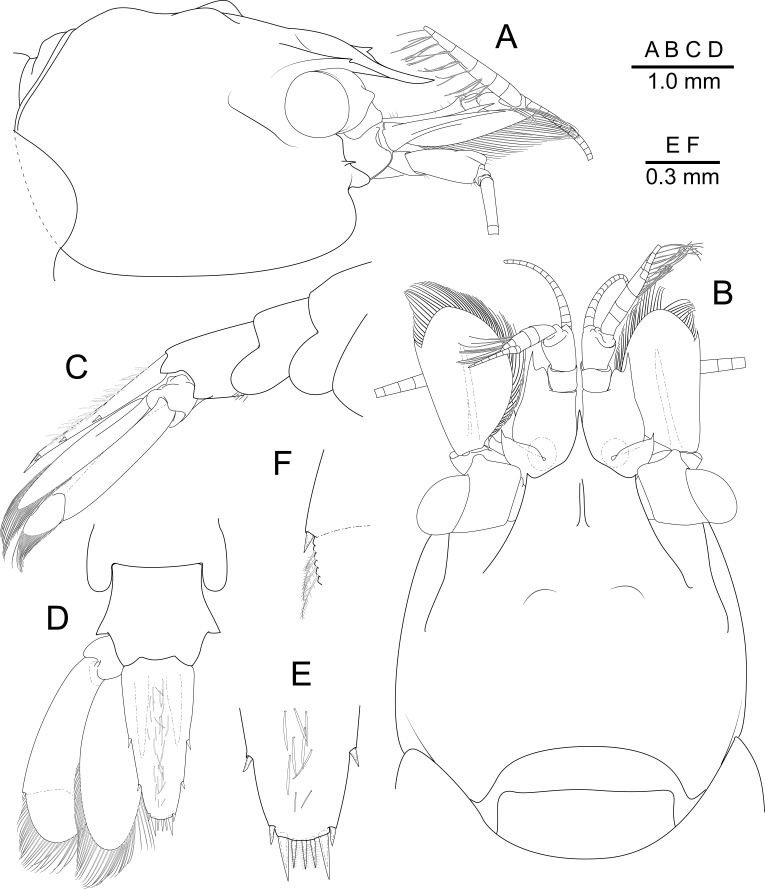
*Pontonidessibogae*, ovigerous female (pocl 2.4 mm) from Munseom Islet, Jejudo Island, Korea (NIBRIV0000896187). **A** carapace and appendages, lateral; **B** idem, dorsal; **C** third to sixth abdominal segments, telson and right uropod, lateral; **D** fifth and sixth abdominal segment, telson and left uropod, dorsal; **E** posterior margin of telson, dorsal; **F** left uropodal exopod, distolateral angle, dorsal.

Pontonides sibogae Bruce, 2005 - Bruce 2005 [377, type locality: Sape Strait, Indonesia, 8°23.5' S 119°4.6' E]; De Grave and Fransen 2011 [370].
Pontonides unciger - Holthuis 1952 [249, figs. 108–112]; Fujino and Miyake 1969 [87, fig. 1]; Heard 1986 [480, Table 1]. (not Calman 1939)
Pontonides cf. sibogae - Marin 2007 [fig. 11C–E].

#### Materials

- **Type status:** Other material. **Occurrence:** recordNumber: JH1109; recordedBy: Jin-Ho Park; Damin Lee; Sang-Hui Lee; individualCount: 1; sex: female; lifeStage: adult (pocl 2.4 mm); reproductiveCondition: ovigerous; preparations: whole animal; dissected (mouthparts, pereiopods) (ETOH); **Taxon:** scientificNameID: urn:lsid:marinespecies.org:taxname:389607; scientificName: *Pontonidessibogae* Bruce, 2005; kingdom: Animalia; phylum: Arthropoda; class: Malacostraca; order: Decapoda; family: Palaemonidae; genus: Pontonides; specificEpithet: *sibogae*; taxonRank: species; nomenclaturalCode: ICZN; **Location:** higherGeography: East Asia; Korea; Jejudo Island; Munseom Islet; waterBody: West Pacific Ocean; island: Jejudo Island; country: Korea; countryCode: Korea/KR; locality: SW Munseom Islet, 33.225092° 126.563375°, 75 m in depth (fcn JH1109); verbatimDepth: 75 m; decimalLatitude: 33.225092; decimalLongitude: 126.563375; **Identification:** identifiedBy: Jin-Ho Park; **Event:** samplingProtocol: Trimix SCUBA diving; eventDate: 2020-01-15; fieldNotes: from a colonial dendrophylliid coral *Dendrophyllia* sp. (leg. DM Lee); **Record Level:** language: kr & en; institutionID: NIBRIV0000896187; collectionID: fcn JH1109; institutionCode: National Institute of Biological Resources (NIBR)

#### Description

Small-sized palaemonid shrimp, body flattened dorsoventrally.

Carapace (Fig. 1A and B) smooth, with two low dorsal tubercles on sides of mid-line, without hepatic tooth. Rostrum short, extending to about 0.8 of first segment of antennular peduncle, toothless, elevated basal crest; lateral carina developed, connecting with supraorbital carina medially. Supraorbital carina well developed, with distinct tooth, expanding to proximal peduncle of eye, forming supraocular eave. Ocular orbit well developed, concave. Inferior orbital angle well-developed, extending ventrad below antennal tooth, roundly produced in lateral view, slightly embracing antennal basicerite. Antennal tooth small and slender, situated along upper margin of inferior orbital angle. Pterygostomial angle roundly produced, non-dentate.

Abdominal pleura of first to fifth somite rounded (Fig. 1C). Sixth abdominal somite about 1.5 times as long as dorsal length of fifth somite. Posterolateral angle broadly subtriangular in shape, pointed, posteroventral angle acute.

Telson (Fig. 1D and E) about 0.6 of pocl, about 2 times longer than maximal width; dorsal surface with numerous fusiform setae longitudinally; two pairs of dorsal spiniform setae, subequal in size, at about 0.5 and 0.8 of telson length, respectively, posterior margin with three sets of spiniform setae, lateral posterior spiniform setae short, about 0.45 of length of intermediate pair, intermediate pair long and stout, smedian pair with three spiniform setae, about 0.6 of intermediate pair length, distally setulose.

Eye (Fig. 1A and B) with ellipsoid cornea, obliquely set on eyestalk, without nebenauge; eyestalk with distinct tubercle anteriorly.

Antennule (Fig. 1A and B) with proximal article of peduncle with convex anterior margin, acute distolateral tooth overreaching intermediate segment, without ventromesial tooth; stylocerite short, sharply pointed, reaching to about 0.4 of proximal article. Intermediate and distal segment short, together equalling about 0.5 of proximal segment length. Upper flagellum biramous, with proximal four segments fused on right side (three on left side), fused part and short ramus with aesthetascs, longer free ramus filiform. Lower flagellum slender, filiform.

Antenna (Fig. 1A and B) with stout unarmed basicerite; carpocerite reaching about 0.7 of scaphocerite; scaphocerite ovoid, about 1.7 times longer than maximal width, acute distolateral tooth situated at about 0.8 of scaphocerite length, exceeded by distal lamella.

**Figure 2.**
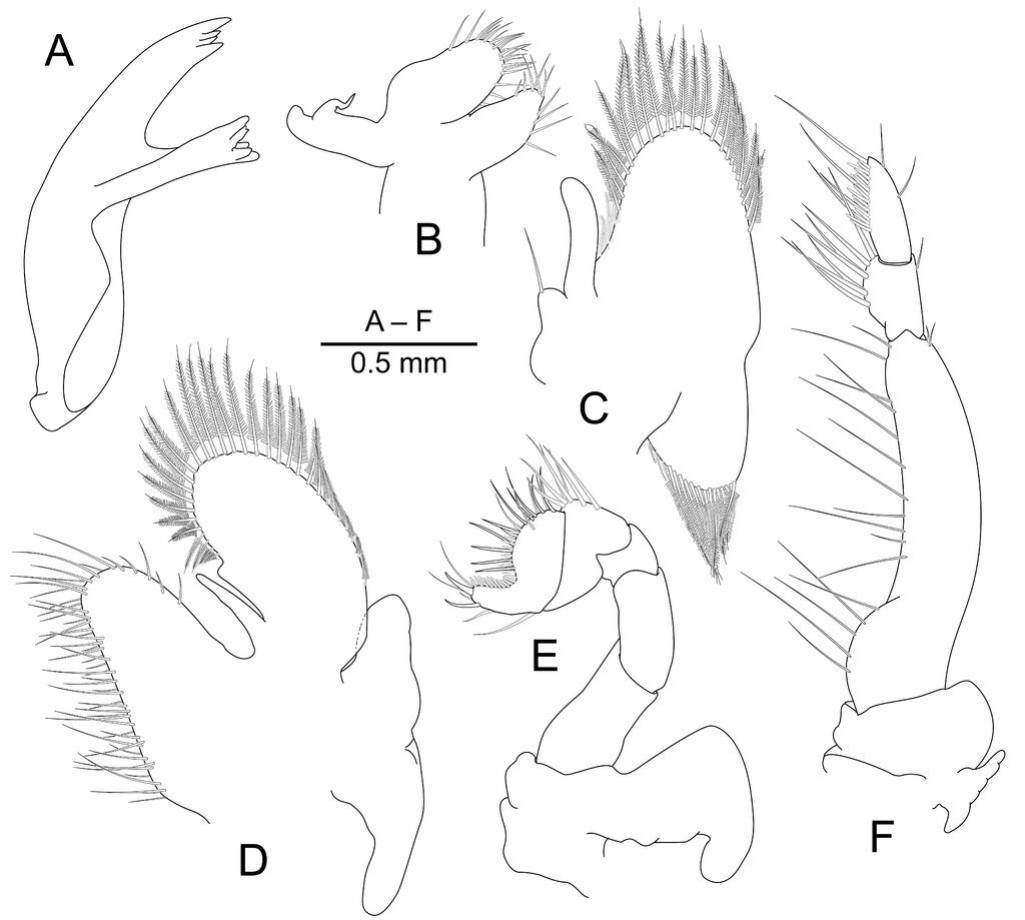
*Pontonidessibogae*, ovigerous female (pocl 2.4 mm) from Munseom Islet, Jejudo Island, Korea (NIBRIV0000896187). **A** mandible, left side; **B** maxillula, left side; **C** maxilla, left side; **D** first maxilliped, left side; **E** second maxilliped, left side; **F** third maxilliped, left side.

Mandible (Fig. 2A) without palp. Molar process robust, with six blunt teeth, without setae. Incisor process with four distal teeth, central pair smaller than outer pair.

Maxillula (Fig. 2B) with bilobed palp. Upper lacinia stout, with six robust spines and several setae distally. Lower lacinia with several setae distally.

Maxilla (Fig. 2C) with tapering non-setose palp. Coxal endite obsolete, basal endite reduced to rounded lobe with mesial margin with long serrulose setae. Scaphognathite normal, about 2.4 times as long as broad, with marginal plumose setae.

First maxilliped (Fig. 2D) with distinct slender palp. Basal and coxal endites fused, with marginal serrulate setae. Exopod with large ovoid caridean lobe, far exceeding palp, with plumose marginal setae. Flagellum of exopod greatly reduced to small knob. Epipod large, widely triangular, bilobed.

Second maxilliped (Fig. 2E) with coxa unarmed, epipod lange, elongate, hooked. Basis without exopod. Propodal segment with simple spines. Dactyl dactylar segment well developed, deeply emarginate medially, with row of stout serrulate spines and simple setae.

Third maxilliped (Fig. 2F) with coxa with rounded lateral lobe, small, but distinct arthrobranch present. Basis completely fused with ischiomerus, about 4.3 times as long as maximal width, with long simple setae medially, exopod absent; lateral margin sparsely furnished with short simple setae. Penultimate segment about 0.24 length of antepenultimate segment, about 1.5 times longer than maximal width, with long and robust simple setae medially. Terminal segment as long as penultimate segment length, about 2 times longer than maximal width, with rows of serrulate and simple long setae medially.

**Figure 3.**
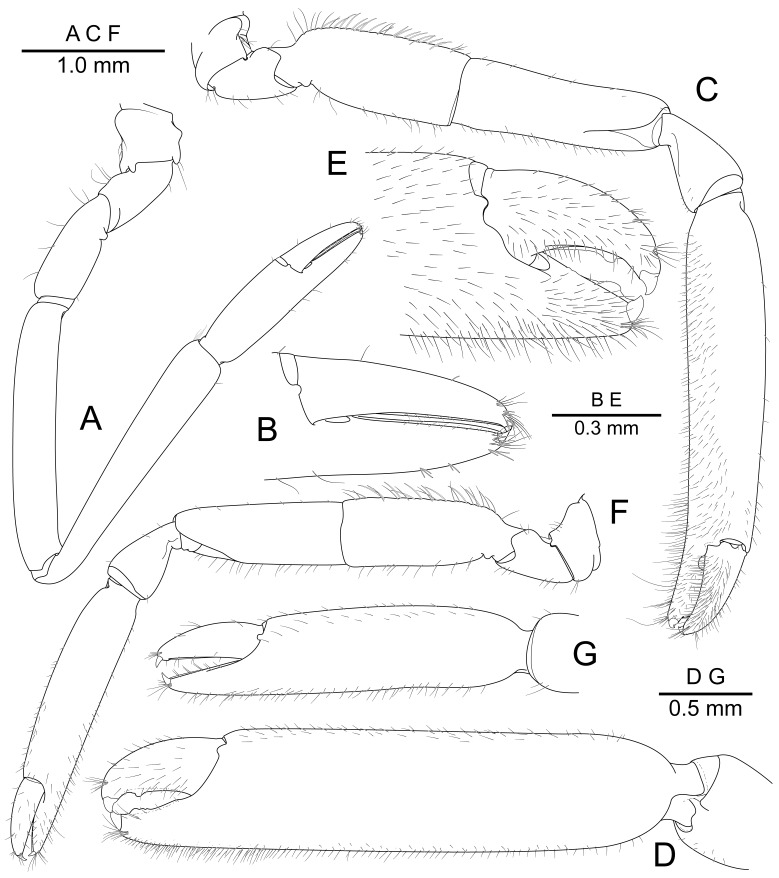
*Pontonidessibogae*, ovigerous female (pocl 2.4 mm) from Munseom Islet, Jejudo Island, Korea (NIBRIV0000896187): **A** left first pereiopod, lateral; **B** idem, distal portion of chela, lateral; **C** right second pereiopod, lateral; **D** idem, distal portion of carpus and chela, mesial; **E** idem, distal portion of chela, lateral; **F** left second pereiopod, lateral; **G** idem, distal portion of carpus and chela, lateral.

First pereiopod (Fig. 3A) coxa and basis without special features. Ischium with simple setae. Merus about 0.95 of carpus length. Carpus about 1.45 times as long as chela, about 7 times as long as distal width, slightly tapering proximally. Carpo-propodal joint with medial cleaning brush. Chela about 4.2 times as long as maximal width, with subcylindrical palm; fingers (Fig. 3B) about 0.8 of palm length, with numerous terminal setae, cutting edges entire, situated mesially, tips hooked.

Second pereiopods similar in shape, unequal in size. Major second pereiopod (Fig. 3C) coxa and basis without special features. Ischium about 0.9 of merus length, with row of fusiform setae along dorsal margin, with simple setae along ventral margin. Merus about 0.6 of palm length, with short simple setae along ventral margin. Carpus about 0.3 of palm length, tapering proximally, unarmed. Chela (Fig. 3D) about 1.1 times as long as pocl, about 2.2 times as long as merus length, with numerous short simple setae along ventromedial margin. Palm cylindrical, smooth, non-tuberculate, about 3.4 times as long as dactylus, about 4.0 times as long as maximum width. Fingers (Fig. 3E) subequal in size, slightly curved mesially, with strong subacute demarcated tip, with simple setae; fixed finger with distinct tooth on cutting edge, large round tooth at about 0.8 of proximal cutting edge; dactylus slightly overreaching fixed finger, about 0.3 of palm length, with distinct tooth at about 0.7 of proximal cutting edge, proximal cutting edge entire.

Minor second pereiopod (Fig. 3F) coxa and basis without special features. Ischium about 0.95 of merus length, with row of fusiform setae along dorsal margin, with simple setae along ventral margin. Merus about 0.75 of palm length, with short simple setae along ventral margin. Carpus about 0.35 of palm length, tapering proximally, unarmed. Chela (Fig. 3G) about 0.65 of pocl, about 1.8 times as long as merus length, with numerous short simple setae along ventromedial margin. Palm cylindrical, smooth, non-tuberculate, about 2.8 times as long as dactylus, about 3.2 times as long as maximum width. Fingers with entire cutting edges, with subacute demarcated tip, with simple setae; dactylus slightly overreaching fixed finger, about 0.35 of palm length.

**Figure 4.**
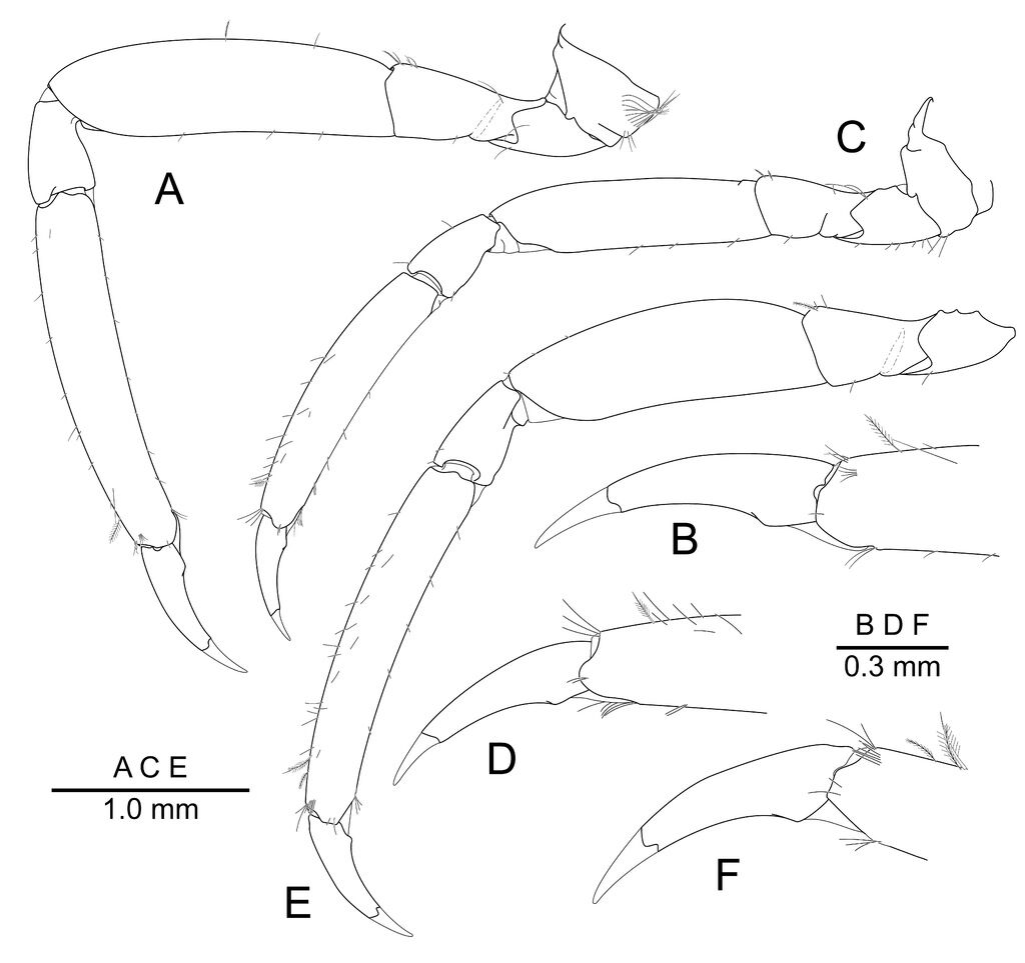
*Pontonidessibogae*, ovigerous female (pocl 2.4 mm) from Munseom Islet, Jejudo Island, Korea (NIBRIV0000896187): **A** third pereiopod, lateral; **B** idem, distal portion of propodus and dactylus, lateral; **C** fourth pereiopod, lateral; **D** idem, distal portion of propodus and dactylus, lateral; **E** fifth pereiopod, lateral; **F** idem, distal portion of propodus and dactylus, lateral.

Ambulatory pereiopods subequal in shape; left fourth pereiopod slightly shorter and more slender than right third and fifth; right ambulatory pereiopods subequal in length. Third pereiopod (Fig. 4A) coxa and basis without special features. Ischium about 0.45 of merus length, unarmed. Merus about 3.1 times as long as carpus length, about 3.8 times as long as maximal depth, unarmed. Carpus about 0.3 of propodus length, about 1.8 times as long as maximal depth, slightly tapering proximally, unarmed. Propodus about 2.2 times as long as dactylus length, about 5.3 times as long as maximal depth, sparsely setose, unarmed. Dactylus (Fig. 4B) about 0.45 of propodus length, about 4.1 times as long as proximal width, uniformly tapering distally, unarmed, with long unguis.

Fourth pereiopod (Fig. 4C) coxa and basis without special features. Ischium, merus and carpus about 0.4, 0.95, 0.35 of propodus length, respectively, unarmed. Propodus about 5 times as long as distal width, about 2.2 times length of dactylus, sparsely setose, unarmed. Dactylus (Fig. 4D) about 3.7 times as long as proximal width, uniformly tapering distally, unarmed, with long unguis.

Fifth pereiopod (Fig. 4E) coxa and basis without special features. Ischium, merus and carpus about 0.35, 0.8, 0.33 of propodus length, respectively, unarmed. Propodus about 6 times as long as distal width, about 3.3 times length of dactylus, sparsely setose, unarmed. Dactylus (Fig. 4F) about 3.4 times as long as proximal width, uniformly tapering distally, unarmed, with long unguis.

Uropods (Fig. 1D) extending well beyond distal margin of telson. Protopodite unarmed laterally. Exopod subequal to exopod length, with lateral border almost straight, entire, terminating in a single mobile spiniform seta, without distolateral tooth (Fig. 1F).

##### Colour

**Figure 5.**
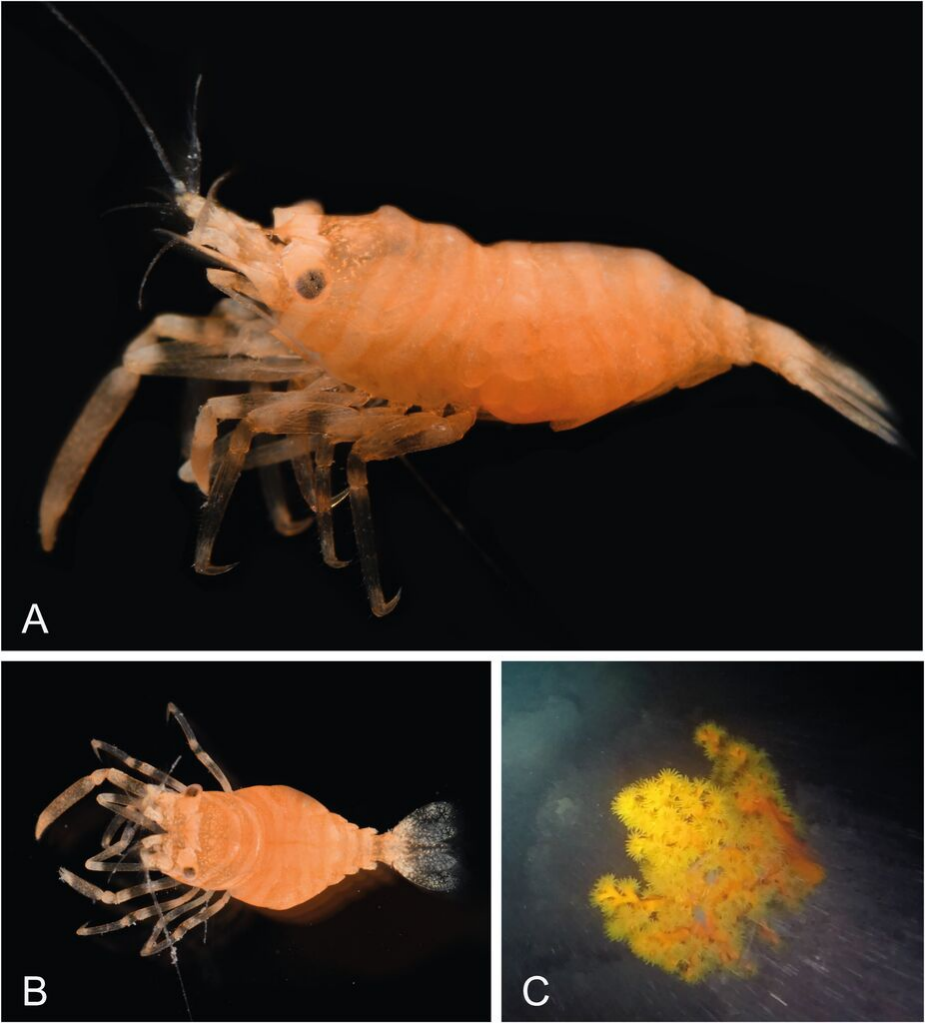
Colour pattern of *Pontonidessibogae* and host invertebrate from Munseom Islet, Jejudo Island, Korea: **A** ovigerous female (pocl 2.4 mm) (NIBRIV0000896187), dorsolateral; **B** idem, dorsal; **C** host colonial dendrophylliid coral. Photographs by JH Park.

The single collected specimen exhibits cryptic colouration in relation to the host species (Fig. 5). Body and appendages generally covered with orange chromatophores, with faint creamy-orange transverse bands from anterior to proximal at regular intervals (Fig. 5A and B). Telson and uropods predominantly transparent with white and yellow chromatophores (Fig. 5B). Pereiopods predominantly creamy-orange colour with translucent bands at regular intervals.

#### Distribution

The species is currently reported in tropical to temperate waters in the West Pacific Ocean, as follows: Indonesia (type locality: Sape Strait, 8°23.5' S 119°4.6' E), Japan (Amakusa Island), and Korea (Jejudo Island) (Holthuis 1952, Fujino and Miyake 1969, present study).

#### Ecology

The present specimen was found in association with a colonial dendrophylliid coral *Dendrophyllia* sp., with an orange coenosarc and orange-yellow tentacles (Scleractinia, Dendrophylliidae) (Fig. 5C). The host coral was only observed between 65 and 75 m depth.

## Discussion

The morphology of the single Korean specimen of *Pontonidessibogae* examined herein corresponds closely to the main diagnostic characters and the illustrations of the holotype (Holthuis 1952), as well as the supplementary descriptions and illustrations in Fujino and Miyake (1969) and Bruce (2005), especially in the presence of a distinct tubercle on the eyestalk, as well as the second pereiopods with fusiform setae along the dorsal border of the ischium. However, some differences between the present material and the illustrated description of the holotype specimen are evident: (1) the carapace has two low dorsal tubercles anterior to the mid-length of the carapace on sides of mid-line (vs. without tubercles in the holotype specimen), (2) the abdominal pleura of the first to fifth somite are rounded (vs. first to fourth somite rounded only, fifth somite with narrow apex in the holotype specimen), (3) the telson has numerous dorsal fusiform setae longitudinally (vs. absence of fusiform seta on telson in the holotype specimen), and (4) the incisor process of the mandible bears 4 teeth (vs. 3 teeth in the holotype specimen). In addition, the present specimen shows a different number in the submedian spiniform setae in the posterior margin of the telson (three in Korean specimen vs. two in holotype). Similar morphological variation has been reported in other species of the genus, such as *P.ankeri* and *P.loloata* and are not considered to be anything, but infra-specific variation.

Holthuis (1952) provided an illustration of the second, right side pereiopod, including the fusiform setae on the ischium dorsal margin. Based on the discussion of sexual dimorphism of the second pereiopod in Fujino and Miyake (1969), especially the relatively short proportion of chela length (including palm) compared to the major second pereiopod, suggests this to be the minor pereiopod. A similar short proportion in the minor second pereiopod is evident on the female specimen from Korea (Fig. 3F). The presence of the fusiform setae has been reported on the mouth parts or pereiopods of all species of *Pontonides* (Holthuis 1952, Fujino and Miyake 1969, Bruce 2005, Marin 2007), in what has been considered to be species specific patterns. The present specimen clearly shows the fusiform setae on the ischium of the second pereiopods as with the previous records (Fig. 3C and D). However, the distribution of them on the major second pereiopod differs from what has been reported. They were described as “present on both the upper and the lower borders of the ischium” by Fujino and Miyake (1969), but in the present material only occur on the upper border. The Korean specimen also has fusiform setae on the dorsal midline of the telson (Fig. 1D and E); which has not been reported previously for *P.sibogae*, nor for the other species of *Pontonides*, but could have been abraded or overlooked.

The present specimen was found in association with the scleractinian coral *Dendrophyllia* sp., similar to the Japanese material in Fujino and Miyake (1969). Marin (2007) documented that *P.maldivensis* is also a scleractinian dendrophyllid associated species. However, *P.sibogae* can be easily distinguished from *P.maldivensis* by the presence of the tubercle on the eyestalk and the fusiform setae on the ischium of the second pereiopods only (vs. without tubercle on the eyestalk and the fusiform setae on the ischium of all five pereiopods in *P.maldivensis*).

## Supplementary Material

XML Treatment for Pontonides sibogae
